# Nanopesticides by Design: A Review of Delivery Platforms, Environmental Fate, and Standards for Safe and Sustainable Crop Protection

**DOI:** 10.3390/molecules31030453

**Published:** 2026-01-28

**Authors:** Yujiao Wang, Zhiwei Tang, Chuhela Tabusibieke, Haixiang Gao, Wei Lu

**Affiliations:** 1 College of Agriculture, Xinjiang Agricultural University, Urumqi 830052, China; yujiaowang@xjau.edu.cn (Y.W.); 18320027289@163.com (Z.T.); c21521sahpr@foxmail.com (C.T.); 2 College of Science, China Agricultural University, West Road of Yuanmingyuan No. 2, Haidian District, Beijing 100193, China

**Keywords:** nanopesticide, delivery platform, environmental fate, ecotoxicology, risk assessment, standards

## Abstract

Nanopesticides are pesticide formulations in which intentionally designed nanoscale carriers shape how an active ingredient (AIng) is deposited, transformed, and released. These systems can improve retention and efficacy, but carrier complexity introduces challenges: nanomaterials can transform in real soil–water matrices, reshaping exposure and risk. These processes are hard to quantify because test protocols and risk assessment frameworks for nanopesticides remain underdeveloped. In this review, we relate design choices across major carrier families—including polymer and lipid particles, nanoemulsions, porous inorganic carriers, and bio-based nanomaterials—to transformations in soil–water systems. We then connect these transformations to ecotoxicological evidence across key non-target taxa. We also address a central “measurement gap” in current risk assessment. Many standard tests were developed for dissolved chemicals. As a result, they do not capture (i) particle stability in realistic matrices, (ii) particle-bound versus dissolved (and ion-released) forms, or (iii) time-resolved exposure. Finally, we propose a Safe-and-Sustainable-by-Design roadmap that prioritizes low-hazard materials, predictable degradation, life-cycle thinking, and staged data generation to enable scalable, field-relevant adoption.

## 1. Introduction

Nanotechnology is increasingly used to reformulate crop protection products. In these systems, the active ingredient (AIng) and/or functional excipients are engineered as nanoscale structures. In this review, we use “nanopesticide” for pesticide products whose performance, environmental fate, or hazard is governed by intentionally designed nanoscale features. Many reported systems are ~1–1000 nm in at least one dimension. Some regulatory definitions focus on a narrower window near ~1–100 nm.

Conventional small-molecule pesticides remain essential for crop protection, but formulation adjuvants and limited control of deposition/release can contribute to off-target dispersion, persistent residues, and non-target exposure. Nano-enabled formulations can reshape this balance by tuning retention and release kinetics; however, their particulate nature introduces matrix-dependent transformations (e.g., aggregation, eco-corona formation, dissolution, and degradation) that complicate environmental fate analysis and risk assessment.

Accordingly, this review aims to establish a unified descriptive framework that links the rational design of nanopesticides with their environmental behavior and risk evaluation. By focusing on key physicochemical parameters—including particle size, morphology, surface chemistry, biodegradability, and stimuli-responsive release properties—we systematically analyze the design principles governing nanopesticide performance, their environmental transformations, and associated ecotoxicological effects. Furthermore, we highlight critical gaps in current evaluation systems and measurement methodologies, with the objective of guiding the safe and sustainable development of nanopesticide technologies.

### 1.1. Limitations of Conventional Pesticides and Advantages of Nanopesticides

Only a fraction of applied pesticide typically reaches and remains at the biological target under open-field conditions. Loss occurs through droplet bounce and runoff, volatilization, UV photolysis, and soil adsorption. Nanopesticides aim to reduce these losses by controlling droplet retention, penetration, and release kinetics.

Conventional emulsifiable concentrates and wettable powders often depend on volatile organic solvents or large surfactant loads to suspend hydrophobic AIngs. This can increase drift and dermal exposure. It can also promote rapid dilution or wash-off under field conditions. Poor aqueous solubility limits leaf uptake and root transport. Photolability and alkaline hydrolysis can shorten residual activity. These factors can prompt over-application and selection pressure for resistance [1]. Foliar delivery is further hampered by the cuticular barrier. Leaf surfaces also contain heterogeneous microclimates that reduce deposition and spreading efficiency [2].

Nanopesticide formulations include polymeric nanoparticles, nanoemulsions, solid lipid nanoparticles, nanogels, mesoporous silica, layered double hydroxides (LDHs), and metal–organic frameworks (MOFs). Encapsulation can raise apparent solubility and protect labile AIngs. It can also improve rainfastness and photostability [3]. Release kinetics can be tuned through diffusion, erosion, or ion exchange [3]. When release aligns with biological windows of susceptibility, similar control may be achieved at lower application rates [3]. Surface ligands and physicochemical tuning (charge, hydrophobicity, and softness) can increase adhesion to epicuticular waxes. They can also promote entry through stomata and increase residence in apoplastic spaces [3,4]. These changes can raise local bioavailability at pest feeding sites [3,4]. Stimuli-responsive carriers can unlock payloads in target microenvironments. Examples include alkaline insect midguts and fungal infection sites [5]. Co-delivery can further support performance and resistance management. A carrier can co-deliver an active ingredient (AIng) with a synergist (e.g., a cytochrome P450 inhibitor) [6,7]. Some formulations also co-deliver multiple AIngs at a fixed ratio [6,7]. Together, these strategies can reduce application frequency and total AIng mass while maintaining or improving control [8,9].

### 1.2. Environmental/Ecological Safety Concerns of Nanopesticides

While nanopesticides can reduce organic-solvent burdens and lower applied AIng mass, their particulate form introduces particle-specific fate and exposure pathways. Mobility and bioavailability can depend on particle behavior, not only on AIng chemistry. Key processes include eco-corona formation, homoaggregation and heteroaggregation, and carrier-mediated redox chemistry. These processes can shift transport, uptake, and toxicity independently of the free AIng. We therefore summarize reported effects in pollinators, soil invertebrates, and aquatic organisms. We also highlight mitigation-oriented design strategies, including controlled degradability and avoiding strongly cationic surfaces.

In natural waters, nanopesticides behave as suspended particles rather than fully dissolved molecules. If particles remain stably dispersed, they can travel farther from the application site. In real waters, particles often stick to clay minerals, natural organic matter, or microbial biofilms. This attachment is known as heteroaggregation. It changes effective particle size and density, which can speed up or slow down settling. These interactions also influence uptake by aquatic organisms and movement up the food chain (trophic transfer) [10,11].

Particles can also acquire surface coatings from the environment. Protein- and polysaccharide-rich coronas can form in soil porewater or on leaf surfaces. These coatings can mask targeting ligands and change biological recognition. They can also alter dissolution rates of inorganic carriers [12,13].

**Figure 1:**
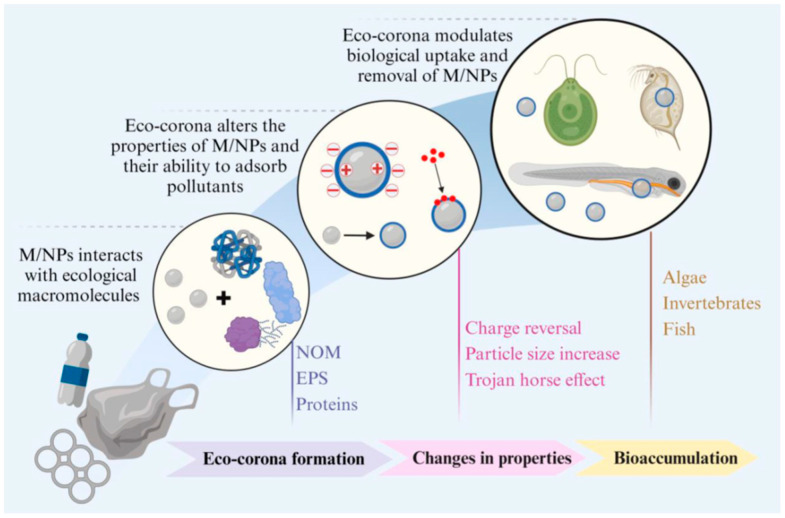
Effects of eco-corona formation on surface properties and bioaccumulation of M/NPs. (Adapted from Yang et al., 2025) [13].

As conceptually summarized in Figure 1, this transformation can give a particle a new “biological identity”. Natural organic matter (NOM), extracellular polymeric substances (EPS), and proteins can adsorb to the surface and form an “eco-corona”. This coating can change surface charge and effective particle size. It can also shift uptake and bioaccumulation patterns in algae, invertebrates, and fish [13].

Some carriers are redox-active or photoreactive. They can generate reactive oxygen species (ROS) and perturb membranes. These processes can cause sublethal metabolic stress in non-target microbiota, pollinators, and aquatic larvae. This can occur even when the free AIng is comparatively benign [14,15]. Degradability is a double-edged sword. Rapid breakdown can release pulses of AIng. Persistent scaffolds may accumulate in sediments or in digestive tracts, with uncertain long-term consequences [16]. Co-formulants can add hazard. Cationic surfactants or quaternary ammonium ligands used for loading can be toxic. They can also alter microbiome composition [17]. Standard mass-based dose metrics (mg L^−1^) can misrepresent exposure for particulate systems. Particle number, surface area, and ion-release flux may better map to biological responses [18]. Finally, chronic low-level exposure could select for behavioral or physiological adaptations in target and beneficial species. This can complicate integrated pest management (IPM) [19]. These concerns do not imply that nanopesticides are inherently unsafe. Instead, they highlight the need for design-for-degradation, realistic characterization under environmental conditions, and test systems that capture particle-specific fate and effects alongside AIng toxicity [16].

### 1.3. Scope and Aim of This Review

This review provides a mechanistic bridge between nanopesticide formulation design and safety evaluation. It is written for readers in formulation chemistry, environmental science, and regulatory toxicology. Rather than cataloging all nano-enabled pesticide formulations, we prioritize systems with clear physicochemical characterization. We focus on systems that also describe a delivery or selectivity mechanism and report evidence relevant to environmental fate, exposure, or non-target effects.

Section 2 surveys delivery architectures, including biomacromolecular payloads and stimuli-responsive systems. It also covers multicomponent co-delivery and emerging carriers such as MOFs, LDHs, and bio-based nanomaterials [20]. Section 3 tracks transformation across soil–water interfaces and within biota [21,22,23]. Section 4, Section 5 and Section 6 then synthesize ecotoxic mechanisms, measurement needs, and a Safe-and-Sustainable-by-Design (SSbD) roadmap for nano-enabled crop protection [24,25]. Where data are available, we highlight quantitative metrics of efficacy and safety. We also provide practical case studies on major crops (e.g., *Glycine max*, *Zea mays*) and relevant pests. For accessibility, Table 1 defines key specialized terms and acronyms used throughout. Finally, Section 7 summarizes the main conclusions and outlines priorities for SSbD-aligned, field-relevant risk assessment and standardization.

We searched Web of Science and Scopus (up to October 2025) using combinations of keywords including “nanopesticide”, “nanoformulation”, “stimuli-responsive”, “environmental fate”, “eco-corona”, and “risk assessment”. We prioritized peer-reviewed studies that report physicochemical characterization of the carrier. We also required a clearly stated delivery or selectivity mechanism and evidence relevant to fate, exposure, or non-target effects. Studies lacking basic characterization or mechanistic linkage were deprioritized.

## 2. Recent Advances in Nanopesticide Delivery Systems

Section 2 outlines delivery strategies that control deposition, penetration, and release through nanoscale design. We organize advances by design logic. We focus on four themes: biomacromolecular delivery, stimuli-responsive release, multicomponent co-delivery, and emerging carrier classes.

### 2.1. Novel Nanopesticides Based on Biological Macromolecules

Biomacromolecular active ingredients, such as double-stranded RNA (dsRNA) and peptides, can be highly selective. However, they degrade quickly and are easily washed off. They also face barriers to foliar entry and cellular uptake. Nanoformulations can help by shielding the payload, improving adhesion, and controlling release. For these systems, the carrier–cargo complex largely determines uptake at pest or pathogen interfaces. We therefore emphasize reporting key quality attributes, such as size distribution, polydispersity, and surface charge. These attributes should be linked to biological endpoints, such as gene silencing or mortality.

Macromolecular active ingredients include dsRNA, small interfering RNA (siRNA), and bioactive peptides. They can shift pest control from broad biochemical inhibition to sequence- or motif-level precision. Field translation depends on several steps. The payload must be protected from nucleases and proteases. It must cross cuticular barriers and reach cells. For RNA interference, it must reach the intracellular RNA-induced silencing machinery. Chitosan nanoparticles, lipid-like (lipidoid) nanoparticles, and dendrimeric polycations can condense dsRNA into ~80–200 nm complexes. These complexes resist RNase degradation and can improve endosomal escape in insect gut epithelia. Such delivery can increase knockdown efficiency at much lower doses than naked RNA [30,31].

LDH “nanoclays” and mesoporous silica can also protect dsRNA. LDHs can shelter dsRNA in anion-exchange galleries, while silica can store it in pores. These structures can provide slow release that matches feeding windows. They can also improve rainfastness on foliage [32]. For systemic delivery to sap-sucking pests, surface chemistry matters. Zwitterionic or PEGylated coatings can reduce nonspecific protein adsorption. This can improve movement through the apoplast and support phloem transport after foliar application.

Peptide-based nanopesticides span two roles. In one role, the peptide is the payload. Examples include insecticidal peptides and antifungal antimicrobial peptides. Encapsulation in solid lipid nanoparticles or polypeptide nanogels can reduce proteolysis and photolysis. In the other role, peptides act as targeting or penetration ligands. They can be grafted onto polymeric or lipid carriers to improve adhesion to leaf waxes, stomatal pores, or pathogen biofilms [33]. Cell-penetrating motifs, such as TAT or R9, can also increase leaf retention and mesophyll entry. This can enable lower spray volumes for comparable pest or disease control [34].

**Figure 2:**
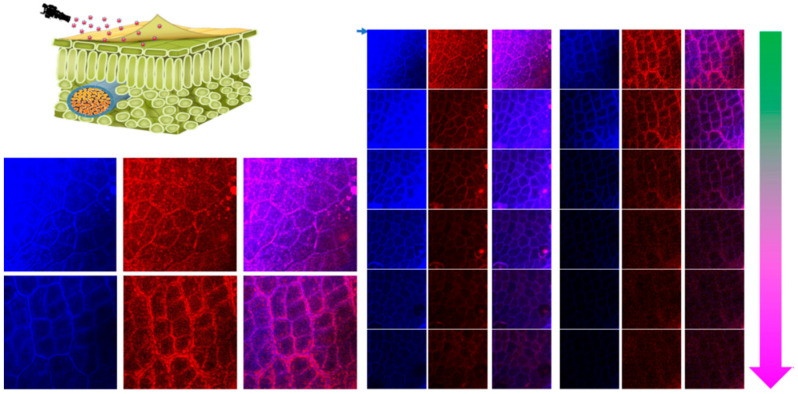
Size-dependent foliar penetration of nano-enabled delivery systems. Confocal Z-stack fluorescence images compare a ~3 nm “unimolecule” formulation with ~111 nm nanoparticles after foliar application. The unimolecule shows deeper penetration into the mesophyll, while the larger nanoparticles remain near outer layers. (Adapted from X. Li et al., 2025) [33].

Foliar entry is strongly size-dependent. The cuticle and the cell wall impose an exclusion limit for many particles. A recent study highlighted the advantage of very small constructs for mesophyll penetration [33]. In Figure 2, a ~3 nm “unimolecule” formulation penetrated deeper into the leaf tissue than ~111 nm nanoparticles. The fluorescence profile extended to a reported ~8 µm depth for the unimolecule system. The larger nanoparticles remained near the surface at a reported ~2 µm depth. Together, these profiles show deeper mesophyll penetration for the ~3 nm construct than for the ~111 nm nanoparticles. This suggests that reducing carrier size can improve delivery of biologicals to intracellular targets [33].

Co-formulation can further improve performance. dsRNA or peptides can be combined with synergists that improve uptake or pathway efficiency. Examples include RNAi pathway boosters and membrane-active amphiphiles. Such combinations can reduce the need for organic solvent-based adjuvants [35]. Key challenges remain for translation. Manufacturing must control size, zeta potential, and polydispersity. These attributes should predict potency under diverse field microclimates. Standardized assays are also needed to separate carrier effects from macromolecule effects in non-target taxa [34,36].

### 2.2. Stimuli-Responsive Nanopesticides

Stimuli-responsive nanopesticides aim to release the active ingredient preferentially at the site of action. They use cues found in the target microenvironment or externally applied inputs. Common triggers include pH, enzymes, redox conditions, and light. Other triggers include temperature, humidity, ionic strength, and magnetic fields. Trigger chemistry must translate into predictable release kinetics. Field translation therefore requires mapping realistic trigger distributions to release profiles.

**Figure 3:**
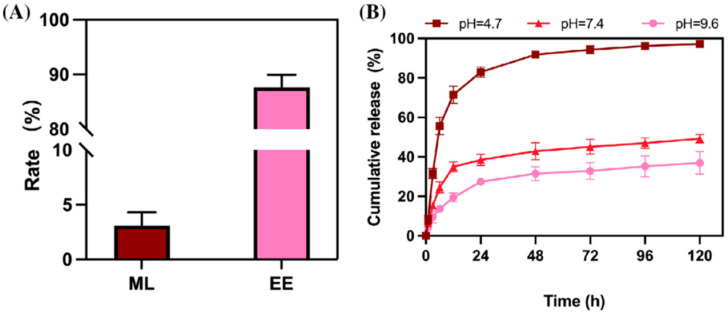
(**A**) Acid-triggered release of matrine from a chitosan-based nanocarrier. (**B**) Cumulative release is shown at pH 7.4 and pH 4.7. The original study used pH 4.7 as an acidic trigger. ML = matrine loading (wt% of carrier). EE = encapsulation efficiency (% of initial matrine encapsulated). (Adapted from Wu et al., 2026) [38].

pH-responsive systems can exploit alkaline insect midguts. Many lepidopteran midguts reach pH 9–11. Carriers built from acid-labile polymers or ion-exchangeable LDHs can remain stable on leaf surfaces. After ingestion, they can dissolve or swell and release the payload rapidly [37,38]. Acid-responsive systems are used in different contexts. Some fungal infection sites and plant defense responses create acidic microenvironments. A chitosan-based carrier developed by Wu et al. (2026) illustrates this approach [38]. In Figure 3, matrine release is faster at pH 4.7 than at pH 7.4 or pH 9.6. This design aims to increase release where disease-related acidity is present and reduce premature release elsewhere [37,38].

Enzyme-responsive designs use cleavable linkers or gated pores. Esterases, chitinases, or pathogen-secreted enzymes can open these gates, aiming to release payloads under target-associated enzyme cues while limiting release under non-target conditions [39]. Redox- and ROS-responsive carriers follow a similar logic. Under the reductive microenvironment associated with fungal infection, disulfide bonds can cleave, enabling rapid release. As an illustrative case study, Liang et al. reported a pesticide delivery system (PRO@DMON-GA-Fe(III)) built on biodegradable disulfide-bridged mesoporous organosilica, in which a gallic-acid/Fe(III) gatekeeper responded to redox changes and enhanced targeted fungicidal activity [40,41]. This example also underscores the need to validate trigger specificity under realistic matrices (e.g., leaf surface films and soil porewater). As an illustrative case study, Liang et al. reported a pesticide delivery system (PRO@DMON-GA-Fe(III)) built on biodegradable disulfide-bridged mesoporous organosilica, where a gallic-acid/Fe(III) complex acts as a gatekeeper. Under the reductive microenvironment associated with fungal infection, disulfide cleavage destabilizes the carrier and accelerates payload release, exemplifying pathogen-triggered redox gating for site-biased delivery [41].

Light-addressable carriers can provide time control that conventional sprays cannot. Photothermal shells, such as polydopamine or carbon dots, can convert light to heat. Photolabile caging groups can also break under light exposure. These approaches can trigger release or improve cuticle diffusion in selected canopy layers [39,40]. Moisture-responsive and ionic-strength-responsive gels can swell under dew or guttation events. This can align release with periods of active feeding. Magnetic colloids can improve retention on leaf undersides when low-intensity fields are applied in high-value crops [41]. Multi-trigger logic can improve selectivity. For example, a carrier can require both a pH change and an enzyme signal. This reduces premature release in open environments [29].

**Figure 4:**
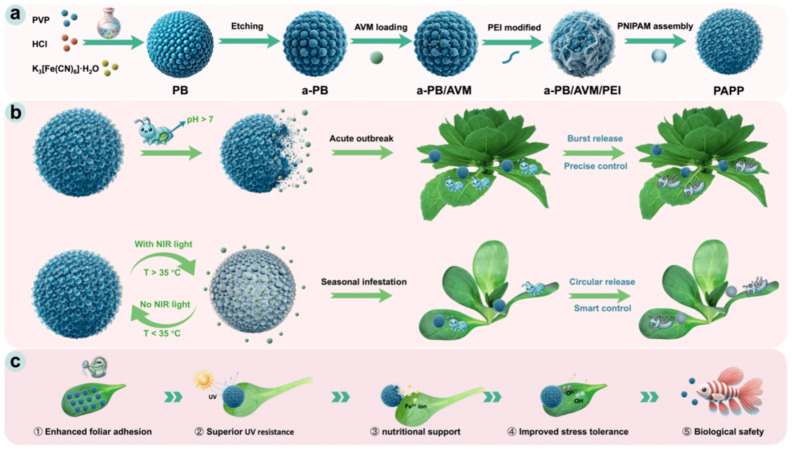
Light-triggered burst and sustained release in a photothermal gating system. (**a**) A near-infrared absorbent (Prussian blue, PB) converts NIR irradiation into heat. Heat drives a phase transition of a thermoresponsive polymer gate (PNIPAM) on mesoporous silica nanoparticles. This enables on-demand release and repeated release cycles. (**b**) Release curves illustrate NIR-triggered rapid release followed by lower-rate release. The original study compared pH 7.4 and pH 9.0 conditions. (**c**) Illustration of the enhanced foliar adhesion, superior UV resistance, nutrient support through Fe^3+^ release, improved crop stress tolerance, and biological safety for PAPP. (Adapted from Teng et al., 2025) [29].

A recent example illustrates multi-input control. Teng et al. (2025) combined Prussian blue cores with a thermoresponsive polymer gate [29]. Their gate material was poly(N-isopropylacrylamide) (PNIPAM). In Figure 4, an alkaline trigger enabled a rapid release mode. Near-infrared (NIR) light produced local heat through the Prussian blue core. This heat opened the gate and enabled repeated release cycles. This design aims to support both rapid knockdown and seasonal maintenance dosing.

Key translational barriers remain. First, trigger intensity and duration must be quantified in real canopies. Second, the trigger response must remain stable over wet–dry and light–dark cycles. Carrier fragmentation into persistent debris must be avoided [42].

### 2.3. Multi-Functional and Multi-Component Nano-Systems

Multifunctional and multicomponent nanosystems aim to address complex pest pressure and resistance evolution. They co-deliver two or more components in one formulation. These components can include multiple active ingredients or actives plus synergists. Co-delivery can preserve an effective ratio at the target site. It can also reduce variability relative to tank mixing. Because these systems are mixtures, efficacy claims should be paired with mixture-aware fate and toxicology assessments. We also note emerging designs that include tracers for deposition measurements.

Some nanopesticides combine two independent ways to control pests in one particle. One common approach is co-delivery of an active ingredient with a metabolic synergist. Examples include inhibitors of P450 enzymes or esterases. This can maintain efficacy at a lower application rate. It can also slow resistance development because pests face more than one selective pressure. Representative designs encapsulate these components in polymer–lipid hybrid nanoparticles. This can preserve a fixed ratio at the pest interface and reduce batch-to-batch variability relative to tank mixes [7,43].

Other systems combine fast-acting and slow-acting components. One payload can provide rapid knockdown, while another provides longer protection. Sequential-release architectures support this timing control. Examples include core–shell particles, Janus particles, and gated mesopores. These designs can tune release to match the temporal order of biological vulnerabilities [44,45]. RNAi–chemical hybrids illustrate a different strategy. dsRNA can silence detoxification or cuticle-formation genes. A reduced dose of a conventional active ingredient can then deliver lethality. This pairing can lower selection pressure for both modalities and restore susceptibility in refractory species [30,46].

**Figure 5:**
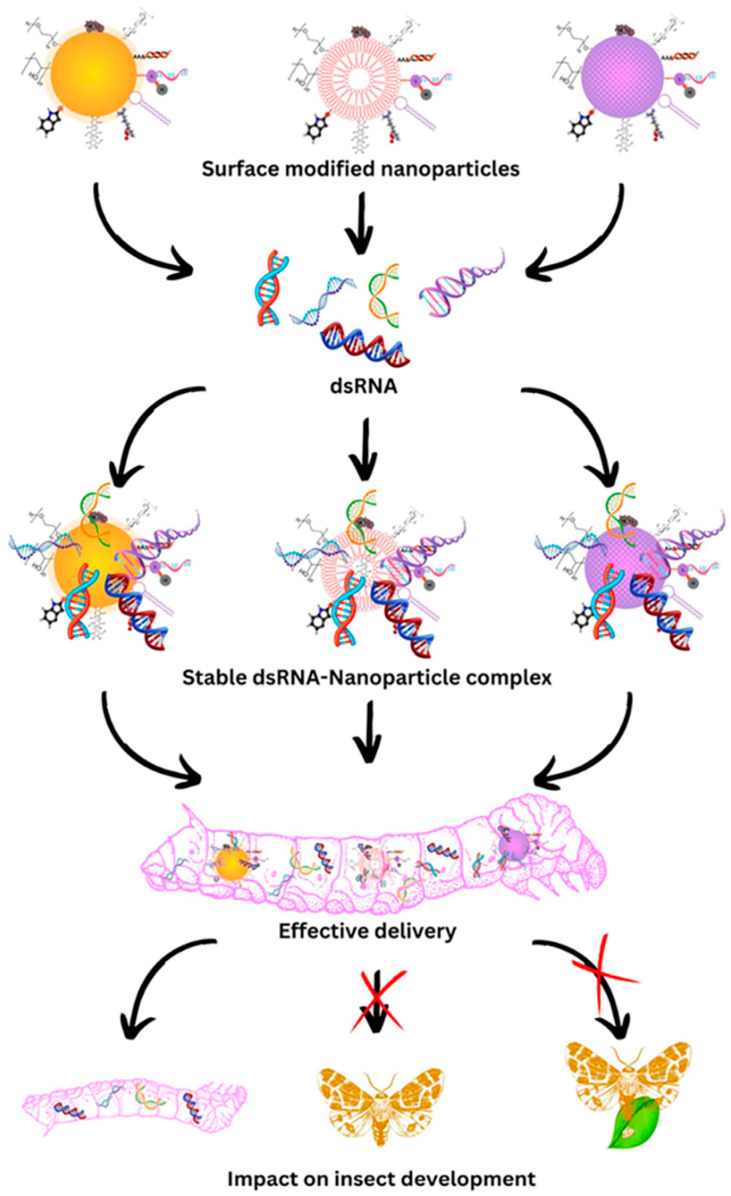
Conceptual workflow for nanoparticle-mediated dsRNA delivery. Surface-modified nanoparticles complex with dsRNA to form a stable construct. This supports delivery to the target insect and leads to impacts on development. (Adapted from Arjunan et al., 2024) [30].

For RNAi hybrids, the carrier is essential. It should protect dsRNA from environmental and biological degradation. It must also deliver dsRNA to the target insect and enable intracellular entry. Figure 5 summarizes this logic. A surface-modified nanoparticle forms a stable complex with dsRNA. This complexation supports delivery to the gut and promotes biological impact compared with naked dsRNA [30].

Multicomponent carriers can also include non-pesticidal components. Some systems incorporate micronutrients or phytohormone primers. Examples include Zn^2+^, SiO_2_, and jasmonate mimics. These additives can prime plant immunity and provide additive crop protection benefits [47,48]. Other systems build adjuvancy into the carrier shell. Amphiphilic adjuvants and wax-affinity peptides can improve rainfastness and leaf residence. This can reduce the need for surfactant sprays that are prone to drift [49]. “Agrotheranostic” constructs add tracers to support deposition monitoring. Fluorophores and carbon dots can map persistence in situ. This can support closed-loop optimization of field application [50].

These systems require quantitative mixture assessment. Synergy should be demonstrated over realistic exposure windows and matrices. Antagonism must also be ruled out. Polymer–active interactions can reduce bioavailability in some cases. Response-surface modeling and isobologram analysis can quantify interactions. Fate-aware metrics, such as surface-area dose and ion-release flux, can improve interpretation [51,52].

### 2.4. Nanopesticides Based on Novel Carriers

Carrier choice governs loading capacity and release mechanism. It also influences degradability and potential carrier-related hazards. Here we introduce emerging scaffold classes, including MOFs, LDHs, and bio-based nanomaterials. We relate their chemistry to release control through pore gating, ion exchange, and framework dissolution. A recurring trade-off is stability versus persistence. High stability supports shelf life and rainfastness. However, it can increase environmental persistence or metal release. Overly rapid degradation can reduce efficacy through premature loss. We connect these considerations to the safety-by-design criteria discussed later.

As a representative example, Liu et al. used a large-pore MOF (PCN-777) to load the bulky macrocyclic lactone AIng abamectin and employed sequential gatekeepers to achieve multi-stimuli responsiveness (pH, laccase, phosphate ions, and phytic acid), illustrating how large-pore MOFs can accommodate complex AIngs and implement multi-signal release control [53]. This design combined robust loading with multi-stimuli responsiveness and reduced premature leakage [54]. From a sustainability perspective, carrier composition matters. Metal choice and ligand degradability should be considered. Metal speciation should also be tracked to avoid accumulation in sediments or food webs [55,56].

LDHs are anion-exchangeable nanoclays. They can intercalate carboxylates, phosphonates, and dsRNA. Release can be triggered by ion exchange and matrix chemistry. LDHs can also adhere to waxy cuticles through charge-mediated interactions [57]. However, real soils contain competing anions and humics. Carbonate and NOM can displace intercalated cargo and cause premature release. Coatings and composition tuning can improve retention under variable ionic strength [16]. Dissolution kinetics should also be balanced. Leachate testing in relevant soils and waters is therefore needed [32].

Bio-based nanomaterials can improve degradability and reduce embodied energy. Examples include lignin nanoparticles, nanocellulose, starch or zein nanospheres, and chitosan or alginate nanogels. Lignin can absorb UV and act as an antioxidant. This can improve photostability and may reduce ROS-related stress. Nanocellulose provides high surface area and strong film formation on leaves [58]. Protein and polysaccharide carriers can also be engineered with enzyme-cleavable crosslinks. This can enable gut- or pathogen-triggered release and reduce persistent residues. These carriers are compatible with solvent-minimized manufacturing. Examples include spray drying and microfluidic flash nanoprecipitation. Such processes can improve batch consistency and reduce cost at agricultural scale [53].

## 3. Environmental Fate and Biotransformation of Nanopesticides

Section 2 focuses on on-target delivery. In Section 3, we follow nanopesticides after deposition, wash-off, or drift into soil and water. Nanopesticides behave as dynamic particles, so their size, surface, and speciation can change quickly. These changes control where particles move, what organisms contact, and how the AIng becomes bioavailable.

### 3.1. Transport, Aggregation, and Sedimentation at the Soil–Water Interface

Nanopesticide mobility in soil and water is controlled by colloid processes. This matters because those processes set the concentrations that non-target organisms actually experience. Here we summarize aggregation, attachment to natural surfaces, settling, and remobilization, using plain descriptors first. A practical implication is that transport parameters should be measured under matrix-relevant conditions.

In natural waters, nanopesticides often behave as suspended particles rather than dissolved molecules. Particles can collide with each other and form larger clusters (homoaggregation). They can also attach to other particles such as clays, metal oxides, or biofilms (heteroaggregation). These processes change particle size and density, which then change settling and transport [13,27].

To model particle movement through soil, clean-bed filtration theory provides a useful starting point for transport in porous media. In the η–α framework, η describes how efficiently particles reach grain surfaces, and α describes how often they attach. However, real soils rarely match the assumptions of ideal filtration. Particles rapidly acquire an eco-corona, which is a coating of natural biomolecules that changes surface interactions [13,27].

Water chemistry strongly controls aggregation and attachment. Multivalent cations such as Ca^2+^ and Mg^2+^ reduce electrostatic repulsion and promote bridging by natural organic matter (NOM). As a result, zeta potential often decreases and aggregation accelerates in circumneutral waters [54]. Heteroaggregation with montmorillonite, iron (oxyhydr) oxides, or biofilm polymers can shift particles into sediments [16].

Unsaturated soils add retention processes that do not occur in saturated lab columns. Particles can be trapped at air–water interfaces by capillary forces. Aggregates can be retained by film straining, meaning physical blockage in thin water films and pore throats [59]. Wet–dry cycles can then release particles in pulses during rain or irrigation events [55].

Field soils are spatially heterogeneous. pH and ionic strength can vary near roots due to root exudates and microbially driven gradients. Therefore, aggregation and deposition can differ across millimeters to centimeters. Single “bulk” measurements can miss these micro-scale hotspots [56].

Design choices made for spray performance can also affect environmental transport. For example, cationic or adhesive surfaces can increase attachment to negatively charged minerals. This can shorten travel distance but increase local sediment burdens [60]. In contrast, NOM-rich eco-coronas can stabilize some particles and promote movement through macropores [61].

Transport is only one side of fate. Chemical transformation can also change what persists and what organisms are exposed to. Some carriers or additives can participate in redox or photo-driven reactions, but this is not universal [23]. Where such pathways occur, they may change both carrier integrity and AIng persistence [23].

**Figure 6:**
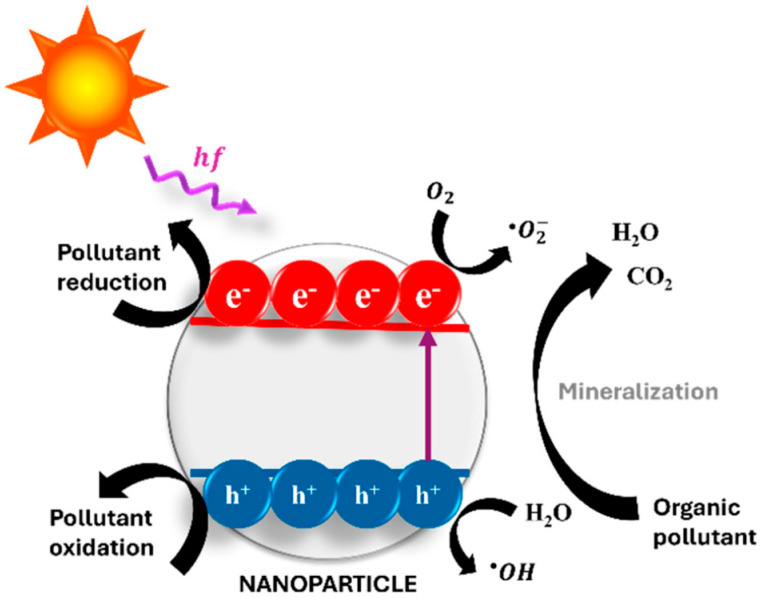
Environmental fate and transformation pathways of nanopesticides in soil–water–plant systems. After application, nanoparticles may aggregate, settle, dissolve, form eco-coronas, and interact with minerals or biofilms, which together control mobility and exposure. Some carriers can contribute to redox- or photo-driven processes that generate ROS. However, these pathways depend on carrier chemistry and environmental conditions, and they should not be assumed universal. (Adapted from Cardoso e Bufalo et al., 2025) [23].

As one example, some semiconductor-like nanomaterials can form ROS under sunlight. This requires suitable electronic structure and sufficient irradiation, and it can be suppressed by scavengers such as NOM [23]. ROS (e.g., •OH, O_2_•^−^, ^1^O_2_, H_2_O_2_) can contribute to oxidative transformation of organic compounds [23]. Therefore, photocatalysis should be treated as a conditional pathway, not a default fate mechanism [23] (Figure 6).

Fate is formulation- and site-specific. Parameters such as aggregation rate, attachment, and settling should be measured in realistic waters and soils. Those parameters then inform exposure in Section 4 and Section 5. In the next subsection, we link transport to AIng release and carrier degradation.

### 3.2. Release Kinetics of AIngs and Degradation of Carrier Materials

Environmental exposure depends on when and how the AIng leaves the carrier. This matters because “slow release” can lower peaks but extend low-level exposure. Here we summarize common release motifs and the field variables that shift them. We then describe how carrier degradation changes both persistence and exposure form.

Release often reflects more than one physical process. For polymeric nanocapsules and nanogels, diffusion and matrix relaxation can both contribute. The Korsmeyer–Peppas model is a common empirical way to distinguish diffusion-controlled from relaxation-controlled release. Humidity, temperature, and surfactants can increase polymer mobility and raise AIng flux [23].

Field water chemistry can speed up ion-exchange release. For LDHs, carbonate and phosphate can outcompete intercalated anions. Therefore, release can be faster in real waters than in low-ionic-strength buffers [28]. This is a frequent reason why lab release curves do not predict field behavior [32].

Porous carriers use gating to regulate diffusion. Mesoporous silica and some MOFs can approach near-constant release when pore entrances are capped. Caps can be polymer brushes or enzyme-cleavable “gates” [62]. However, repeated wet–dry cycles can fatigue caps and shift kinetics toward burst-then-tail release [62].

Lipid carriers can change permeability through phase behavior. Solid lipid nanoparticles can undergo polymorphic transitions that alter packing and diffusion pathways. Lipases and temperature can accelerate those transitions in biological or wet leaf environments [63]. Therefore, leaf wetness events can amplify release relative to dry conditions.

Carrier degradation can reduce long-term particulate persistence, but it can also create pulses. Polyesters and polysaccharides can hydrolyze or undergo enzymolysis to smaller oligomers [59]. If erosion is faster than diffusion, the AIng can be released in a short time window. This is beneficial for efficacy in some cases, but it can increase transient off-target exposure [59].

**Figure 7:**
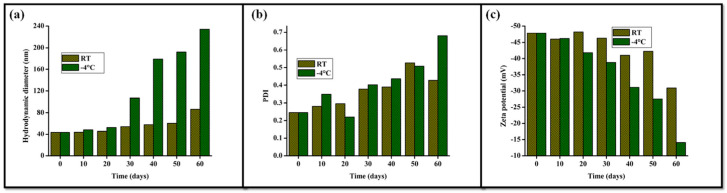
Storage stability of a carvacrol nanoemulsion (CAR-NE) stabilized by polysaccharide-based surfactants. Hydrodynamic diameter (**a**), PDI (**b**), and zeta potential (**c**) change over time and depend on temperature, illustrating that colloidal integrity can be an early and sensitive indicator of formulation persistence. (Adapted from Choudhary et al., 2024) [59].

Colloidal destabilization often appears before complete chemical breakdown. For “green” nanoemulsions, stability can be highly sensitive to storage and temperature. In one example, hydrodynamic size increased and zeta potential moved toward neutrality during cold storage [59]. Such changes can alter both spray performance and environmental mobility (Figure 7).

Inorganic scaffolds primarily degrade by dissolution. Amorphous silica can dissolve to monosilicic acid, and LDHs can leach metals during structural change [32]. MOFs can degrade by metal–ligand hydrolysis, with rates controlled by pH and ligand chemistry. Eco-coronas can also slow or accelerate dissolution by changing local chemistry at the surface [32].

Photochemical processes can either protect or accelerate AIng transformation. Some shells can scavenge radicals and reduce oxidative loss of the AIng. Other shells can promote ROS generation under light and increase degradation rates [64]. Thus, the net effect depends on shell composition and realistic light spectra [64].

For exposure assessment, an “AIng half-life” alone is often insufficient. Risk should track the effective release half-life (t_1/2,rel_), which combines release and degradation. This metric is more relevant to time-dependent exposure than bulk dissipation curves [65,66]. It also links directly to the testing concerns in Section 5.

Key implication: Release and degradation are coupled. They are strongly shifted by water chemistry, humidity, light, and wet–dry cycling. Therefore, release tests should use realistic matrices and time-resolved sampling. Next, we discuss how plants and microbes further modify nanopesticide fate.

### 3.3. Uptake, Translocation, and Transformation in Biota (Plants, Microorganisms)

Once particles contact organisms, biological barriers control uptake and internal exposure. This matters because apparent “accumulation” can reflect surface binding rather than true internalization. Here we separate adsorption, compartmental retention, and internal transport in plants and microbes. We also highlight how transformation products can matter for risk assessment.

Plant exposure begins at leaf and root surfaces. On leaves, stomata and microcracks are possible entry points, but waxy cuticles limit transport. Surface chemistry controls whether particles penetrate or remain bound to the cuticle. Moderately hydrophilic, near-neutral surfaces often penetrate more than strongly cationic shells that bind tightly [4].

**Figure 8:**
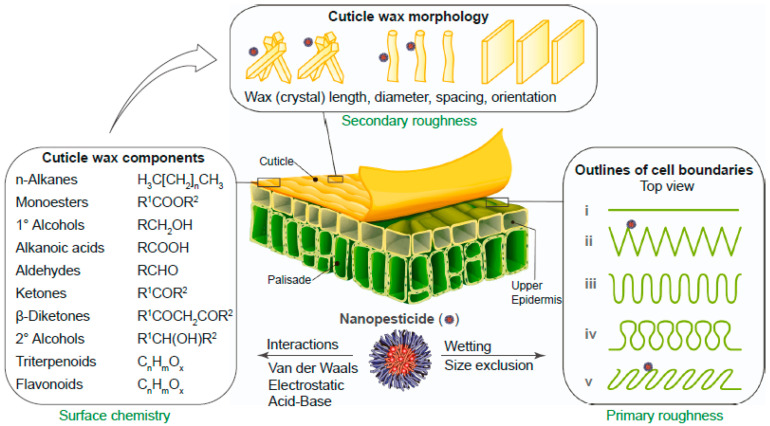
Schematic of the plant cuticle interface. The cuticle has hierarchical roughness and chemically diverse wax components, which together shape wetting, adhesion, and size-dependent transport of foliar nanopesticide. (Adapted from Arcot et al., 2024) [4].

Leaf surfaces are physically complex. Epicuticular wax crystals create micro- and nano-scale roughness that affects wetting and adhesion. This roughness can amplify size exclusion and make penetration strongly size-dependent [4]. Therefore, carrier size and surface affinity should be described together, not separately (Figure 8).

After entry, transport route depends on size and aggregation. Particles with low aggregation propensity can move apoplastically into xylem flows. Larger or adhesive constructs often stall in the cell wall matrix [67]. As a result, “systemic movement” claims require evidence of both particle integrity and location.

Phloem delivery faces additional constraints. For sap-sucking pests, phloem mobility is often required for efficacy. Zwitterionic or PEGylated coatings can reduce interactions with phloem proteins and improve movement [68]. In other cases, carrier degradation may release a phloem-mobile AIng, enabling transport as molecules.

Biotransformation can occur in plants and in their associated microbiomes. Plant esterases, glycosidases, and redox environments can transform both carriers and AIngs [69]. For dsRNA payloads, plant Dicer processes dsRNA into siRNAs that can traffic systemically. This fate route does not exist for conventional small-molecule AIngs [69].

Microbial interactions are often decisive for fate in soil and on leaves. In the rhizosphere and phyllosphere, EPS in biofilms can trap particles [70]. This reduces mobility but can increase contact with enzymes that erode carriers [71]. Therefore, biofilms can both localize exposure and accelerate transformation.

Microbes can detoxify or activate compounds. Bacteria and fungi can transform AIngs by oxidation–reduction and hydrolysis. They can also change inorganic scaffolds by releasing metal ions during degradation. Thus, transformation products should be considered when interpreting ecological effects [72].

For RNAi-based nanopesticides, microbial degradation is a practical efficacy barrier. Environmental nucleases can reduce dsRNA persistence before it reaches the target [30]. At the same time, unintended gene silencing in beneficial microbes is a theoretical concern. Current evidence suggests low risk, but data outside model taxa remain limited [30].

**Figure 9:**
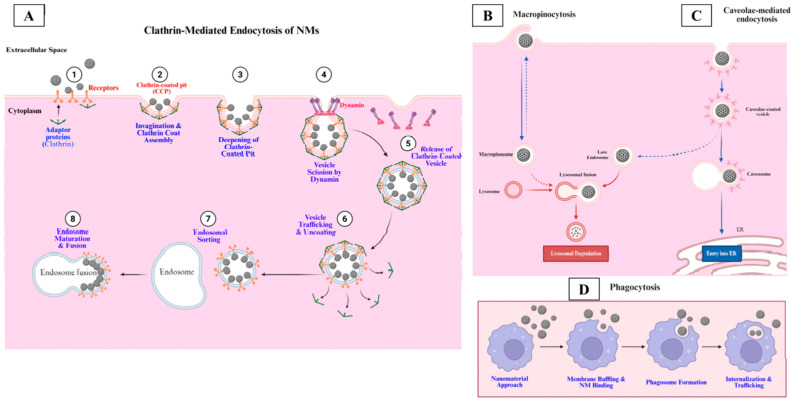
Primary endocytic pathways for nanoparticle uptake. Corona composition can shift which uptake routes dominate and therefore alter intracellular trafficking and degradation. Cellular level uptake of NMs through (**A**) clathrin-mediated endocytosis of NMs in insects, (**B**) Macropinocytosis, (**C**) Caveolae-Mediated Endocytosis, (**D**) Phagocytosis. (Adapted from Kamalakannan et al., 2025) [73].

Eco-corona evolution can change how cells recognize and internalize particles. The eco-corona acts as the particle’s “outer surface” that interacts with receptors. Changes in corona composition can shift endocytosis pathways and intracellular trafficking [73]. Therefore, time-resolved corona characterization can help predict biological fate in situ [73] (Figure 9).

Biological processing reshapes nanopesticide fate after application. Adsorption, transport, and biotransformation depend on size, surface chemistry, and eco-coronas. These processes determine internal exposure and therefore influence toxicity interpretation. Next, Section 4 summarizes evidence for off-target effects across taxa.

## 4. Ecotoxicological Effects of Nanopesticides

Section 4 focuses on off-target receptors, not on pest control. This matters because hazard can arise from the AIng, from the carrier, or from their interaction as a mixture. We organize evidence by taxa, then by mechanisms, and finally by longer-range outcomes such as trophic transfer. Throughout, we emphasize how to separate AIng-driven toxicity from carrier-driven effects.

### 4.1. Toxicity to Non-Target Organisms

Non-target effects depend on exposure route, exposure duration, and effective dose at biological interfaces. This matters because nanopesticides can change all three even when nominal AIng dose is unchanged. We summarize evidence for pollinators, soil biota, and aquatic organisms receiving runoff. We also emphasize that studies should report matrix conditions that control aggregation and corona formation.

**Pollinators.** Nanoformulations can change both uptake and residence time in the gut. For example, nanoemulsions and polymeric carriers can increase oral uptake and prolong gut exposure. This can shift LD_50_ values and increase sublethal outcomes such as impaired foraging and navigation [4]. Contact exposure can also increase when cationic shells adhere strongly to cuticle and setae [4].

Carrier chemistry can affect pollinator microbiomes. In honeybees, ingestion of some carriers has been linked to shifts in dominant gut symbionts. These shifts can occur even when the AIng is considered relatively “bee-safe” [17,74]. Therefore, microbiome endpoints may be informative for chronic and sublethal assessment.

**Soil biota.** Soil organisms experience particles in pore water and in ingested soil. In *Eisenia fetida* and springtails (*Folsomia candida*), growth and reproduction effects have been linked to ROS and membrane stress [75,76]. Organic matter can form eco-coronas that partially passivate surfaces and reduce apparent potency [77,78]. Thus, soil composition should be treated as a key experimental variable and reported explicitly [75,76].

Nematodes highlight why mass concentration can mislead. Some studies report behavioral changes at particle-number doses below mass-based no-effect levels [78,79]. This suggests that particle-based metrics may better capture interface-driven effects [70,79]. It also reinforces that the carrier is part of the toxicological mixture.

**Figure 10:**
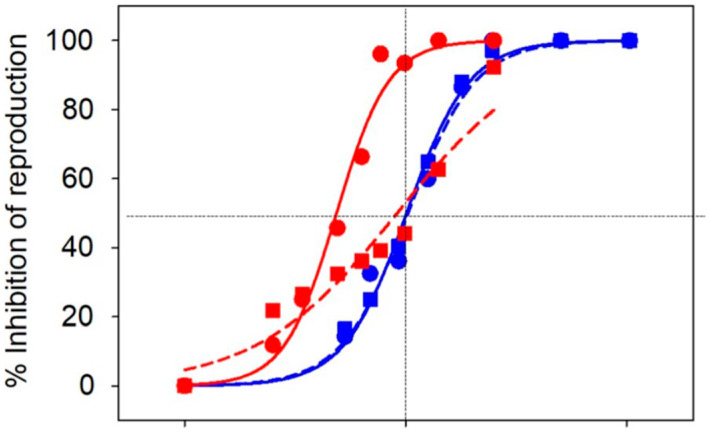
Example of mixture behavior in nanopesticide toxicity. Observed effects can match additive predictions (carrier + AIng) or exceed them when carrier–AIng interactions produce synergy. (Adapted from Eghbalinejad et al., 2024) [70].

Mixture behavior can be additive or synergistic. In *C. elegans*, one nanoformulation’s toxicity was consistent with additive effects of carrier plus AIng. Another formulation showed greater toxicity than expected from simple addition, indicating interaction effects [70]. Therefore, risk evaluation should test the formulation, not only the free AIng and the “empty” carrier [70] (Figure 10).

**Aquatic organisms.** Runoff delivers both dissolved AIng and carrier-bound AIng to water bodies. Some nanoformulations can maintain dissolved AIng near organisms through corona-mediated retention. Others may deliver carrier-bound AIng via ingestion, which can increase gut-localized exposure [80,81]. These pathways can increase immobilization in Daphnia or Chironomus compared with solvent controls [80,81].

Photoactive components can add conditional hazards. Fish embryos and larvae have shown developmental effects under sunlight-mimicking spectra with some photoreactive carriers. These interactions may be absent in dark controls; therefore, studies should report light conditions explicitly [77]. Across taxa, hazard ranking depends on carrier charge, hydrophobicity, and degradability, not only AIng identity [78].

Nanoformulations can change exposure kinetics and exposure route. Therefore, studies should report both dissolved-AIng and particle-associated exposure where possible. They should also distinguish AIng effects from carrier effects using appropriate controls. Next, we summarize mechanism pathways that help interpret these outcomes.

### 4.2. Nano-Property-Mediated Toxic Mechanisms

Mechanisms help determine whether a nanoformulation is safer or simply shifts exposure timing. This matters for design choices in SSbD and for selecting the right test metrics. We summarize three recurring mechanisms: membrane interactions, ROS generation, and ion- or metal-driven chemistry. We then discuss dose metrics that better align with these mechanisms.

Cationic surfaces can directly perturb membranes. Quaternized polysaccharides and PEI-like architectures can increase membrane permeability. This can elevate intracellular Ca^2+^ and trigger mitochondrial ROS even before AIng release [82]. Such patterns support a membrane-initiated stress mechanism.

**Figure 11:**
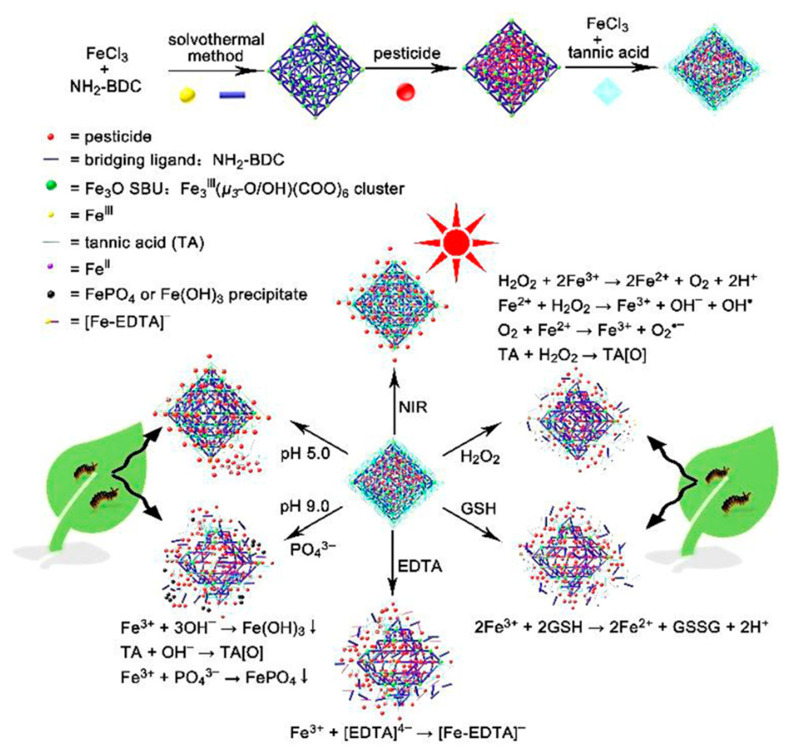
Example of carrier-mediated redox chemistry. An iron-based MOF can generate hydroxyl radicals via a Fenton-like reaction in the presence of H_2_O_2_, illustrating a potential carrier-driven mechanism. (Adapted from Dong et al., 2021) [84].

Some inorganic and hybrid carriers can catalyze redox reactions. Residual metal sites on mesoporous silica or MOFs can promote ROS generation under oxygen and light [83]. In iron-containing frameworks, a Fenton-like pathway can generate hydroxyl radicals in the presence of H_2_O_2_ [84]. This is a carrier-driven mechanism that can occur independently of the Aing (Figure 11).

Not all carriers increase oxidative stress. Lignin nanoparticles can scavenge radicals and quench singlet oxygen. In some studies, this reduced oxidative biomarkers in non-target organisms while maintaining efficacy [85]. Thus, carrier chemistry can either amplify or attenuate oxidative pathways.

Trigger-responsive designs can shift where AIng is released. If enzyme-cleavable linkers respond mainly in pest midguts, external release can be reduced. However, off-target triggers can exist in soil or water, so trigger realism must be tested [86]. This is why Section 5 emphasizes trigger-aware exposure characterization.

Dose metric choice affects mechanistic interpretation. Mass concentration (mg L^−1^) can obscure interface-driven processes. For many particles, surface area or particle number correlates better with ROS generation and membrane damage [87]. For dissolving carriers, ion-release flux can explain toxicity plateaus when dissolution saturates [87].

Mechanistic biomarkers can help separate nanospecific pathways from AIng mode of action. Omics readouts often identify ER stress, transporter upregulation, and innate immune signaling. These signatures can differ from classical AIng pathways and support adverse outcome pathway mapping [88]. Such approaches remain emerging but can strengthen causality when paired with exposure metrics.

Photo-dependent interactions are an additional complexity. Photothermal shells can raise local temperature under sunlight and increase permeability and stress. Experiments that decouple light and temperature can reveal additive or synergistic terms [89]. Therefore, light spectra and irradiance should be documented in photoreactivity-relevant studies.

To summarize, carriers are not always inert. Membrane activity, ROS chemistry, and ion release can each drive hazard. Therefore, nano-specific dose metrics and mechanistic endpoints are often needed. Next, we discuss how these processes influence bioaccumulation and trophic transfer.

### 4.3. Bioaccumulation and Trophic Transfer

Bioaccumulation cannot be judged from single-species acute tests alone. This matters because particles can be ingested and retained without true tissue assimilation. Here we define “pseudo-accumulation,” discuss when vector effects are plausible, and outline implications for food webs. We conclude with practical measurement needs for risk assessment.

Many organisms ingest particles that remain in the gut lumen. In filter feeders and grazers, carrier-bound AIng can appear “high” in whole-body burdens because gut contents dominate. However, tissue assimilation may be limited unless the carrier dissolves or releases AIng intracellularly [90]. Therefore, apparent biomagnification factors can reflect pseudo-accumulation rather than true internal burden.

Vector effects can still occur under some conditions. Particles can sorb hydrophobic AIngs from the surrounding medium and later deliver them after ingestion. Eco-coronas can increase ingestion by changing surface properties and “food-like” cues [91]. Thus, particles can act as shuttles that alter chemical activity at biological interfaces.

**Figure 12:**
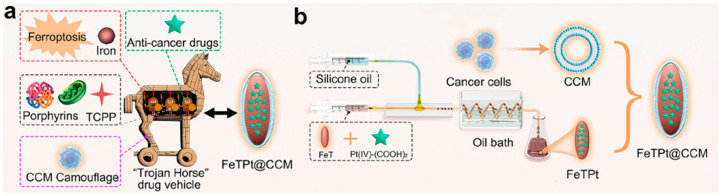
(**a**) the preparation of the FeTPt@CCM and its capability of synergistic chemotherapy, PDT, and ferroptosis therapy. (**b**) The design of the multi-bioinspired “Trojan Horse” drug vehicle. (Adapted from Q. Zhang et al., 2023) [92].

This concept resembles a “Trojan horse” mechanism, but the environmental analogue is the eco-corona. In nanomedicine, coatings can increase cellular uptake of a carrier. In the environment, eco-coronas can similarly increase ingestion or uptake of particles [92]. Therefore, corona formation can influence both exposure route and dose localization [92] (Figure 12).

Laboratory microcosm studies—small, controlled experimental ecosystem mimics that incorporate relevant matrices and multiple interacting organisms to approximate food-web processes—report different outcomes depending on carrier degradability. Rapid carrier dissolution can reduce particle transfer through food webs; however, dissolution can increase the flux of dissolved AIng to higher trophic levels in some cases. Thus, “less particle transfer” does not automatically mean “less chemical exposure” [93].

Terrestrial food webs add recycling pathways. Egested fecal pellets can concentrate intact carriers in detrital pools. This can create re-exposure of soil microbes and detritivores over time [10]. In NOM-rich soils, coronas can accelerate depuration and shorten gut residence.

Within pollinator colonies, trophic dynamics differ from single-bee tests. Larval exposure can be driven by stored food such as bee bread. If carrier persistence is shorter than larval development time, colony-level transfer may decrease [94]. This provides a design target that links degradation to biological timescales [94].

In conclusion, trophic outcomes depend on dissolution, corona evolution, and gut retention. Risk assessment should distinguish particle presence in gut contents from tissue assimilation. It should also track both particle-bound and dissolved AIng along food webs. Next, Section 5 explains why current standards often fail to measure these states reliably [95].

## 5. Challenges in Risk Assessment and Evaluation Methodologies for Nanopesticides

Risk assessment requires that exposure be measurable and reproducible. This matters because nanopesticides change exposure state over time and across matrices. In this section, we focus on practical hurdles: separating particle-bound and dissolved fractions, maintaining stable exposures, and modeling time-dependent behavior. We also outline feasible upgrades that connect directly to SSbD in Section 6.

### 5.1. Analytical and Characterization Challenges in Complex Matrices: Extraction, Identification, and Quantification

Mass concentration alone is insufficient to describe exposure. Depending on the system, organisms may respond to particles, dissolved AIng, or released ions. A practical measurement strategy must therefore track three things: dispersion state, phase partitioning, and time dependence. We summarize common artifacts, then describe a minimum workable method set.

Agricultural matrices are complex and often heterogeneous. Relevant examples include leaf surfaces, dew, rhizosphere pore water, irrigation canals, and bee bread. These matrices contain salts, clays, and biomolecules that drive eco-corona formation and aggregation [96]. Therefore, sample handling can change the very property being measured.

Handling steps can bias size and number estimates. Freeze–thaw cycles can induce aggregation and change apparent size distributions [96]. High-g centrifugation can remove soft aggregates and lower measured particle number [96]. Membrane filtration (0.2–0.45 μm) can selectively retain aggregates and strip coronas [96].

Dilution can also create artifacts. Diluting into low-ionic-strength media may “stabilize” suspensions in the lab. However, this removes heteroaggregation processes that dominate mobility in real waters [97]. As a result, DLS can report smaller diameters and lower polydispersity than in situ.

No single tool is sufficient, so combined methods are needed. Asymmetric flow field-flow fractionation (AF4) separates particles by hydrodynamic size without a stationary phase. When coupled to multi-angle light scattering (MALS) and ICP-MS, AF4 can resolve polydisperse mixtures and track inorganic components [98]. However, recovery losses and membrane interactions must be quantified with matrix-matched spikes.

**Figure 13:**
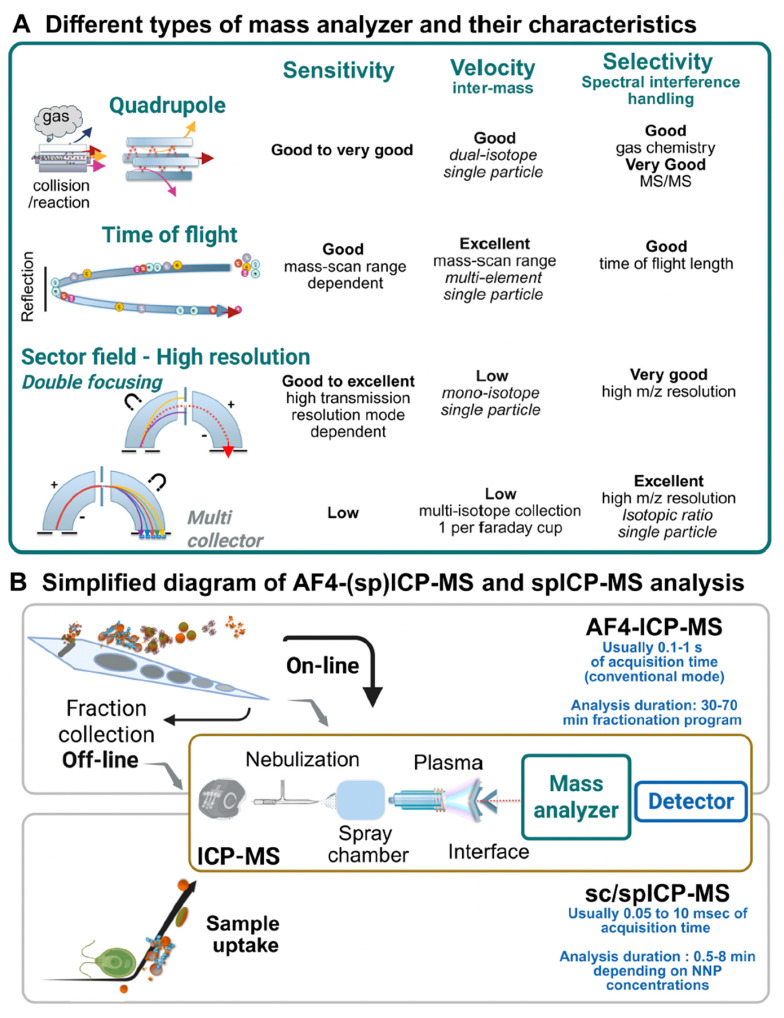
Simplified diagram of AF4-(ICP-MS) and spICP-MS workflows. AF4 provides size-based fractionation, and ICP-MS provides elemental detection, enabling size-resolved elemental distributions when recovery is controlled. (Adapted from Cuss et al., 2025) [99].

Single-particle ICP-MS (spICP-MS) can size and count individual metal-containing particles. This is useful for metal-bearing carriers and doped tracers, including some LDHs and MOFs [99]. However, spICP-MS does not directly detect purely organic carriers without added labels. Therefore, method choice depends on carrier composition and labeling strategy [99] (Figure 13).

Imaging can validate primary morphology and gating structures. Cryo-TEM and cryo-SEM can reduce dehydration artifacts compared with conventional TEM [100]. However, throughput is low and image selection bias is a concern [93]. Thus, imaging should be linked to number-weighted statistics from solution methods.

Separating particle-bound AIng from dissolved AIng requires operational definitions. Common approaches include ultrafiltration, dialysis, centrifugal separation, and AF4 cut points [101]. After separation, LC–MS/GC–MS can quantify the AIng, while ICP-MS/XRF can quantify inorganic carriers [99]. Mass balance often fails unless co-formulants and surfactants are included in extraction workflows.

Labels can help, but they introduce their own problems. Fluorophores can report deposition and integrity, but dye leaching and photobleaching can bias results [102]. Stable isotope labels can be more robust for tracing across matrices and trophic steps. Isotope-dilution quantification can also correct for recovery losses [102]. A major gap is the lack of matrix-appropriate reference materials. Few certified materials mimic eco-corona-coated or weathered nanopesticides. Therefore, interlaboratory comparisons can show large variation for the same sample. This limits regulatory comparability and model calibration [103].

A practical minimum analytical set for dossiers should be feasible in many labs. It can include (i) an AF4-based fractionation workflow, (ii) a particle number method for relevant carriers, and (iii) chemical AIng quantification [103,104]. It should also include validated phase separation steps and QA/QC with matrix spikes [103,104]. When possible, isotope-based corrections should be used to report recoveries and uncertainty [103,104].

Overall, matrix-matched workflows that preserve coronas and aggregation states are more informative than simply adding more instruments. Exposure should be reported as time-resolved partitions between particle-bound and dissolved forms. Next, we explain why current test guidelines often miss these dynamics.

### 5.2. Limitations of Current Toxicological Testing Standards

Most guideline tests were built for dissolved chemicals. This matters because nanopesticides can evolve during the test, shifting between particle and dissolved states. Here we explain the key assumption failures and propose feasible upgrades. We emphasize time-resolved exposure characterization for slow-release systems.

Standard aquatic tests often specify nominal mg L^−1^ concentrations. They typically assume stable exposure over fixed durations. For nanopesticides, exposure can change through aggregation, settling, dissolution, and corona evolution [28]. Therefore, identical nominal doses can represent different biological drivers across laboratories.

**Figure 14:**
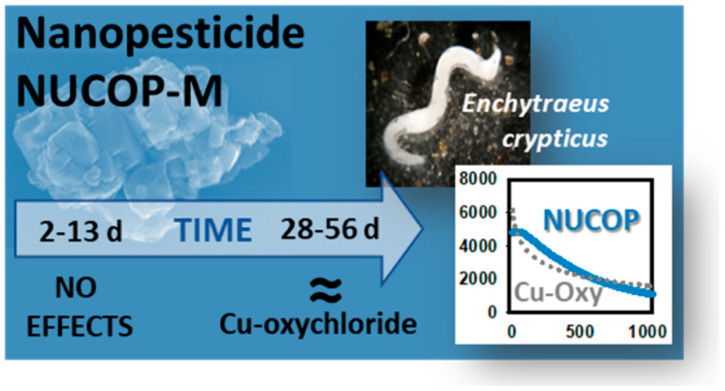
Comparative toxicity of a nanopesticide (NUCOP-M) versus its conventional active ingredient (Cu-oxychloride) over time in *E. crypticus*. (Adapted from the graphical abstract in Chidiamassamba et al., 2024) [28].

Particle-based exposure metrics are rarely required in legacy guidelines. Examples include particle number, surface area, and ion-release flux. Without such metrics, it is difficult to compare studies or identify mechanistic drivers [28]. This is one reason why nano-enabled systems can appear inconsistent across reports. Time dependence can invert hazard conclusions. In one example, a slow-release copper formulation appeared less toxic in short assays (Figure 14) [28]. Over longer periods, toxicity converged with the conventional product as ions accumulated. Therefore, slow release can be misread as “safer” if test duration is too short [28].

Renewal designs can also change the exposure state. Each media change can remove dissolved AIng and reset eco-corona composition. This can reduce realism for particulate formulations and complicate interpretation [34]. Flow-through designs can help, but they are not commonly required [34].

Pollinator guidelines capture key routes but miss colony-level dynamics. Adult and larval OECD tests address oral and contact exposure. However, they do not fully represent storage and provisioning pathways that drive chronic colony exposure [105]. Sublethal endpoints such as microbiome composition and navigation are often optional [105].

Soil guideline tests face similar realism limits. Earthworm and collembolan tests measure growth and reproduction, which are relevant endpoints. However, soil ionic strength and organic matter strongly control heteroaggregation and bioavailability [101]. If these are not controlled or reported, reproducibility and field extrapolation suffer.

OECD TG 318 on dispersion stability is a useful anchor for nanomaterials. However, it does not fully address multicomponent pesticide formulations with adjuvants. It also does not specify how to separate carrier and AIng contributions when both drive hazard [26]. Therefore, nanopesticides need TG-compatible upgrades that remain workable for registrants [26].

Two upgrades are both feasible and high impact. First, embed exposure characterization modules in each relevant TG. At minimum, measure dissolved AIng mass balance and at least one particle-based metric at multiple time points [100]. Second, use trigger-aware scenarios for stimuli-responsive systems so tests reflect intended-use conditions [100].

Endpoints should also match plausible nano-specific mechanisms. When membrane perturbation or ROS pathways are suspected, include relevant biomarkers. When microbiome disruption is plausible, include community structure or function endpoints. Such additions help separate carrier-driven effects from AIng receptor-mediated toxicity [28,106].

In conclusion, current standards can misclassify slow-release and evolving exposures. Feasible upgrades focus on time-resolved characterization and trigger realism. These upgrades also improve cross-lab reproducibility. Next, we discuss exposure modeling and dose–response interpretation.

### 5.3. Complexity in Environmental Exposure Modeling and Dose–Response Relationship

Field risk depends on predicted environmental concentrations and realistic dose–response relationships. This matters because nanopesticides cannot be modeled as a single dissolved chemical curve. Here we explain why models must treat the formulation as a coupled carrier–AIng system. We also discuss how non-monotonic responses and mixture interactions complicate interpretation.

Standard multimedia fate models perform well for many dissolved pesticides. However, they often omit colloid filtration, heteroaggregation kinetics, and corona evolution. They also omit time-dependent release from carriers [54]. Therefore, they can mispredict where and when exposure occurs for particulate systems. Hybrid models are needed for nanopesticides. They should couple particle transport (attachment, detachment, deposition, resuspension) with AIng kinetics (release, degradation, partitioning). They should also include dissolution and competitive ion exchange for metal-bearing or ion-exchange carriers [62]. Such coupling links measurable parameters to predicted exposure states [54].

Canopy microclimate can convert a steady dose into pulsed exposure. Leaf wetness, irradiance, and temperature vary strongly over time and space. These variables can shift release rate constants and transform particles during and after application [107]. Therefore, time-series weather inputs are often necessary for realistic modeling [107]. Uncertainty must be handled explicitly rather than ignored. Formulations are polydisperse, and environmental matrices vary by site. Monte Carlo or Bayesian approaches can propagate uncertainty into predicted environmental concentrations (PECs) [107]. This helps identify which parameters most constrain risk predictions [107].

**Figure 15:**
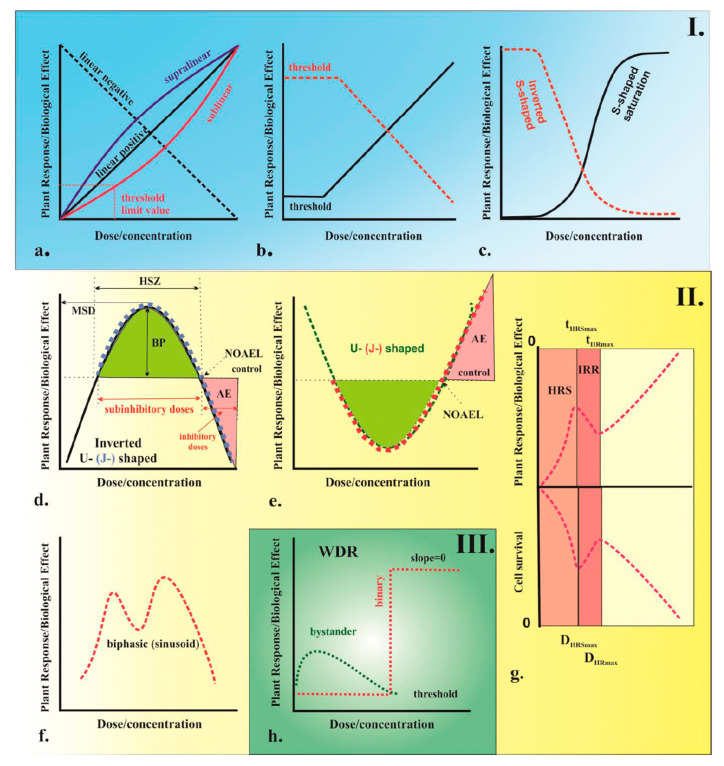
Hypothetical non-monotonic dose–response curves. Such patterns can arise when bioavailability and interface interactions change with aggregation, corona evolution, or dissolution. Hypothetical curves of the dose–response relationship. (**I**). Monotonic type (blue background). (**a**) Linear, sublinear and supra-linear curves with no clear threshold. The lowest dose at which an effect can be observed is named the threshold limit value (TLV); (**b**) threshold curves; (**c**) S-shaped curves (saturation curve). The S-shaped curves are characterized by an increasing trend, reflecting the beneficial effects on the plant organism as the dose/concentration gradually increases. (**II**). Non-monotonic (yellow background). (**d**) U-shaped or J-shaped curves; (**e**). inverted U- or β-shaped curves. This type of curve reflects the hormetic response at low doses; (**f**) biphasic curve; (**g**) N-shaped dose–response curves of HRS/IRR response in terms of DSBs and cell survival. (**III**). Curves without dose–response relationship (**h**). (Adapted from Georgieva & Vassileva, 2023) [108].

Dose–response relationships can be non-monotonic in particulate systems. Low particle numbers may increase membrane interaction, while higher numbers may aggregate and reduce bioavailability [108]. Such patterns can produce U-shaped or inverted U-shaped curves. In these cases, a single EC50 can be misleading if a monotonic model is forced onto non-monotonic data [108] (Figure 15).

Mixture behavior adds another layer of complexity. Formulations include the carrier, the AIng, and often adjuvants. Interaction terms can arise under light or temperature triggers, or through carrier-AIng binding [109]. Response-surface designs can quantify synergy or antagonism more reliably than single-factor tests [109].

Bioaccumulation modeling should separate gut retention from tissue uptake. Compartment models can include “gut lumen” and “tissue” states for particulate and dissolved AIng. Field data streams from AF4 and spICP-MS can constrain rate constants that are otherwise under-identified. This supports more realistic predictions of trophic transfer [110].

In conclusion, modeling must track particles and molecules together. Time-dependent behavior is often the central feature, not a secondary detail. Non-monotonic and mixture responses should be treated as real possibilities, not anomalies. Next, Section 6 translates these insights into SSbD priorities and a staged roadmap [111].

## 6. Future Perspectives

This review links nanopesticide design to environmental fate, exposure, and standards. This matters because safety assessment must follow the formulation as a coupled carrier–AIng system. Looking ahead, we summarize the main progress, identify recurring gaps, translate SSbD into measurable design targets, and propose a staged roadmap that prioritizes feasible near-term actions.

### 6.1. Summary of Current Progress and Key Knowledge Gaps

We highlight three recurring knowledge gaps. First, design parameters are not yet quantitatively linked to in situ release under real weather and canopy microclimates. Second, matrix-dependent fate remains incompletely understood, including eco-corona effects, heteroaggregation, and dissolution. Third, available methods still offer limited mechanistic resolution for separating carrier-driven hazards from AIng hazards in non-target organisms. Closing these gaps is essential to translate promising laboratory demonstrations into robust, safe field products.

Nanopesticide development has moved beyond simple solubilization aids. Many systems now program deposition, penetration, and release through carrier design. Examples include dsRNA and peptide delivery, stimuli-responsive carriers, multicomponent hybrids, and emerging scaffolds. These approaches can reduce solvent burdens and lower effective application rates [112]. Environmental science now supports a more mechanistic view of exposure. Eco-corona formation and matrix-dependent aggregation explain why mobility is site specific [113]. Fate models increasingly treat release as a kinetic parameter rather than a fixed assumption. These advances help connect formulation design to predicted environmental concentrations [113].

Ecotoxicology is also moving toward mechanism-based interpretation. Studies increasingly separate AIng toxicity from carrier-driven membrane, ROS, or microbiome effects. Omics readouts and adverse outcome pathway mapping can support causal links when paired with exposure data. However, standardized exposure characterization remains a limiting step in many studies [112,113,114].

Three knowledge gaps recur across the literature. First, trigger intensity and release kinetics are rarely mapped under real canopy microclimates. Second, fate is often measured in simplified media that do not reproduce eco-coronas and heteroaggregation. Third, carrier-driven hazards are not consistently separated from AIng hazards across taxa [99,112,113,114].

In essence, the field has strong design innovation but weaker cross-study comparability. Progress now depends on time-resolved exposure metrics and matrix-relevant testing. These needs motivate SSbD as a set of measurable constraints. Next, we translate SSbD into actionable design rules.

### 6.2. Application of the “Safe-And-Sustainable-by-Design” (SSbD) Concept in Nanopesticide R&D

SSbD should be treated as an engineering constraint, not a slogan. This matters because formulation choices can shift both efficacy and off-target exposure. Here we propose practical SSbD rules that align with known mechanisms and measurement capabilities. We emphasize degradability, benign chemistry, and bounded release behavior.

First, prioritize carrier building blocks with low intrinsic hazard. This can include GRAS-like chemistries or nutritionally benign elements where appropriate. Second, design degradation to non-persistent products on biologically relevant timescales. These timescales should consider pollinator larval development and aquatic emergence windows [115]. Third, avoid surface chemistries known to drive membrane damage. High cationic surface density is a recurring driver of lysis-like interactions and oxidative stress. Fourth, minimize co-formulants that perturb beneficial microbiomes. These choices reduce carrier-driven hazard independent of AIng identity [115]. SSbD targets should be multi-axis rather than single-metric. Efficacy can be reported as ED_90_ or an equivalent agronomic endpoint. Fate and exposure targets can include effective release half-life (t_1/2,rel_) and residence time in nectar, pollen, and bee bread [116]. For metal-containing carriers, set a bounded ion-release flux that stays below ecotoxic thresholds.

Model-informed screening can accelerate SSbD decisions. Digital twins can couple canopy microclimate, colloid transport, and release kinetics to predict PECs. Bayesian calibration to field measurements can turn uncertainty into an explicit design variable [117]. This supports formulation triage before expensive field campaigns [117]. Life-cycle assessment should run in parallel with formulation R&D. It should account for embodied energy, solvent use, and end-of-life persistence [116]. Continuous, solvent-lean manufacturing can improve batch consistency and lower environmental footprint [116]. Renewable feedstocks can further support sustainability goals when performance is maintained [116].

Finally, SSbD becomes actionable when linked to measurable constraints. Those constraints should reflect known hazard mechanisms and realistic exposure timescales. They should also be compatible with feasible analytical workflows. Next, we provide a staged roadmap to operationalize these upgrades.

Evidence requirements should scale with novelty and persistence. This matters because a one-size-fits-all standard is unlikely to be feasible. We propose a staged roadmap that separates near-term actions from mid- and long-term infrastructure needs. The goal is to improve safety assessment without blocking innovation.

### 6.3. Three-Tier Roadmap

The staged roadmap prioritizing feasible near-term actions is as follows.

Tier 1 (near-term; implementable within existing frameworks).

- Adopt a minimum reporting set: size distribution, zeta potential, AIng loading, release profile, and a matrix-relevant stability or dissolution screen.
- Embed basic exposure characterization in ecotoxicity tests: dissolved-AIng mass balance plus at least one particle-based metric at multiple time points.
- Harmonize terminology and define acronyms at first use, supported by Table 1 and a short abbreviations list.

Tier 2 (mid-term; method development and interlaboratory validation).

- Validate robust workflows to separate particulate versus dissolved fractions in key matrices (e.g., soil pore water, plant tissues, pollen, and nectar).
- Develop matrix-appropriate reference materials and run interlaboratory comparisons to quantify recovery, bias, and detection limits.
- Create trigger-aware scenarios for stimuli-responsive systems so tests match intended-use conditions.

Tier 3 (long-term; infrastructure and policy evolution).

- Establish field-scale monitoring that couples particle-based exposure metrics with ecological endpoints across representative crops and climates [118].
- Build open, shared datasets linking formulation descriptors/design parameters to fate and hazard outcomes to support model calibration and cross-study comparability.
- Evolve regulatory decision frameworks to evaluate nanoformulations as coupled carrier–AIng systems, including time-dependent behavior and mixture effects [10].

Overall, this roadmap turns nanospecific complexity into measurable design space. Tier 1 improves reproducibility now. Tier 2 builds comparability across labs. Tier 3 enables field realism and regulatory modernization.

## 7. Conclusions

Nanopesticides can improve crop protection by increasing retention and precision delivery, but the same nanoscale features also control environmental fate and exposure. Across carrier families, matrix-driven transformations (eco-corona formation, heteroaggregation, dissolution, and degradation) determine when and where AIngs become bioavailable and whether carrier-driven hazards emerge.

Current fate and ecotoxicity studies often lack time-resolved exposure characterization and consistent separation of dissolved versus particulate fractions, limiting cross-study comparability and mechanistic interpretation. A practical SSbD approach therefore requires linking formulation descriptors to measurable constraints—such as AIng loading and release half-life, matrix-relevant stability/dissolution behavior, and benign degradation products—together with minimum reporting and mass-balance checks. The three-tier roadmap proposed here prioritizes Tier 1 implementable reporting and exposure characterization within existing test frameworks, Tier 2 interlaboratory-validated workflows and reference materials, and Tier 3 field-scale monitoring and data-sharing infrastructure to support regulatory modernization. Together, these steps can support innovation while improving confidence in environmental safety assessments.

## Figures and Tables

**Table 1:** Glossary of key terms and acronyms used in this review.

Term/Acronym	Definition
LDH(s)	Layered double hydroxide(s); lamellar “anionic clays” with exchangeable interlayer anions. Used as ion-exchange nanocarriers for anionic AIngs and biomacromolecules (e.g., dsRNA).
MOF(s)	Metal–organic framework(s); porous crystalline coordination materials built from metal nodes and organic linkers. Enable high loading and tunable, stimulus- or degradation-controlled release.
ROS	Reactive oxygen species (e.g., •OH, O_2_•^−^, H_2_O_2_) relevant to oxidative stress and to redox- or photo-driven transformation processes.
AF4 (asymmetric flow field-flow fractionation)	A stationary-phase-free method that separates nanoparticles/colloids by hydrodynamic size. It is often coupled to detectors such as MALS or ICP-MS.
spICP-MS (single-particle inductively coupled plasma mass spectrometry)	Detects and sizes individual metal-containing nanoparticles. It also quantifies particle number concentration.
OECD TG (e.g., TG 318)	OECD Test Guideline(s); standardized protocols for chemical/ecotoxicological testing. TG 318 addresses dispersion stability of nanomaterials in water [26].
SSbD	Safe-and-Sustainable-by-Design; a framework that embeds safety, degradability, exposure, and life-cycle constraints early during material/formulation design [24,25].
GRAS	Generally Recognized as Safe; used here to denote carrier building blocks with established low intrinsic hazard, supporting safer formulation choices.
Application-rate reduction	Achieving comparable or improved efficacy at a lower applied mass of AIng per hectare, enabled by improved delivery/retention and/or synergistic co-delivery.
Eco-corona	A dynamic coating of environmental biomolecules (e.g., NOM, EPS, proteins) that adsorbs to nanoparticle surfaces in real matrices. It can change particle size/charge, colloidal stability, transport, and biological interactions [13,27].
NUCOP-M	Name used for a nano-enabled copper oxychloride formulation exhibiting slow ion-release kinetics relative to the conventional product [28].
Solvent-based adjuvants	Solvent-based adjuvants/co-formulants: organic solvent–containing additives used to solubilize active ingredients (AIngs) or enhance penetration.
η-α framework	Notation from clean-bed filtration theory. η is collector efficiency (transport to collectors), and α is attachment efficiency (sticking probability). The product η·α governs predicted deposition in porous media.
Burst release/sustained (cyclic) release	Burst release: an initial rapid liberation of AIng. Sustained (cyclic) release: maintenance-mode release achieved via repeated stimulus cycles (e.g., photothermal heating and PNIPAM gating; PNIPAM = poly(N-isopropylacrylamide); NIR = near-infrared) [29].
AIng(s)	Active ingredient(s); the pesticide compound(s) responsible for biological efficacy (often referred to as the “active substance” in regulatory contexts). AIng/AIngs is used in this review to avoid abbreviation ambiguity.
Microcosm (study)	Small, controlled experimental ecosystem mimic (often including relevant environmental matrices and multiple interacting organisms) used to investigate fate, exposure, bioaccumulation, and/or trophic transfer under semi-realistic conditions.

## Data Availability

No new data were created or analyzed in this study. Data sharing is not applicable to this article.
